# Efficacy of Intravitreal Brolucizumab for Chronic Central Serous Chorioretinopathy: A Pilot Study

**DOI:** 10.3390/jpm15090409

**Published:** 2025-09-02

**Authors:** Sunjin Hwang, Rim Kyung Hong, Eun Hee Hong, Min Ho Kang, Yong Un Shin

**Affiliations:** Department of Ophthalmology, Hanyang University Guri Hospital, College of Medicine, Hanyang University, Guri 11923, Republic of Korea; sunjin1989@hanyang.ac.kr (S.H.); flarud1201@hanmail.net (R.K.H.); ehhong@hanyang.ac.kr (E.H.H.); bsdoc@hanyang.ac.kr (M.H.K.)

**Keywords:** chronic central serous chorioretinopathy, Brolucizumab, pachychoroid

## Abstract

**Background/Objectives:** Chronic central serous chorioretinopathy (cCSC) is a vision-threatening disorder characterized by persistent subretinal fluid (SRF). While several treatment options exist, their efficacy varies, and optimal management remains uncertain. This retrospective pilot study aimed to evaluate the efficacy and safety of intravitreal brolucizumab in patients with symptomatic cCSC without pachychoroid neovasculopathy (PNV). **Methods:** In total, 15 eyes of 15 patients diagnosed with symptomatic cCSC without PNV were treated with a single intravitreal injection of brolucizumab. Patients were followed for six months. Primary outcomes included resolution of SRF and changes in central subfield thickness (CST) and subfoveal choroidal thickness (SCT). Best-corrected visual acuity (BCVA) and ocular safety profiles were also assessed. **Results:** Complete SRF resolution was observed in 14 of 15 eyes (93.3%) within six months. Mean CST significantly decreased from 317.13 ± 73.40 µm to 205.53 ± 20.17 µm (*p* < 0.001), and mean SCT from 475.87 ± 107.66 µm to 390.13 ± 121.67 µm (*p* < 0.001). BCVA improved in 12 eyes (80.0%) and remained stable in 3 eyes; however, the mean improvement (logMAR 0.34 ± 0.33 to 0.14 ± 0.13) was statistically significant (*p* = 0.007). No significant ocular adverse events were reported. **Conclusions:** Intravitreal brolucizumab may be an effective and safe treatment for reducing SRF and choroidal thickness in patients with cCSC without PNV. Larger, controlled studies are needed to validate these findings.

## 1. Introduction

Central serous chorioretinopathy (CSC) is a retinal disorder within the pachychoroid disease spectrum, characterized by serous neurosensory detachment, diffuse decompensation of the retinal pigment epithelium (RPE), and choroidal dysfunctions such as hyperpermeability and increased choroidal thickness [1]. While many cases resolve spontaneously, approximately 10% of patients develop a chronic course with persistent subretinal fluid (SRF), leading to progressive RPE damage, photoreceptor atrophy, and an increased risk of secondary complications such as pachychoroid neovasculopathy (PNV) [2]. Studies utilizing indocyanine green angiography (ICGA) and enhanced depth imaging (EDI) optical coherence tomography (OCT) have provided insights into the underlying choroidal vascular abnormalities, revealing dilated and congested choroidal vessels along with increased choroidal thickness in eyes with CSC [3].

The treatment landscape for chronic CSC has evolved over the years. While thermal laser photocoagulation has been largely abandoned due to potential collateral damage, various pharmacologic interventions—including mineralocorticoid receptor antagonists (eplerenone, spironolactone), rifampin, and acetazolamide—have been explored with varying degrees of success [4,5,6]. Photodynamic therapy (PDT) with verteporfin has emerged as the most effective treatment by inducing choroidal vascular remodeling and reducing hyperpermeability [7,8]. However, PDT remains off-label, carries the risk of choroidal hypoperfusion, and has become less accessible due to a global shortage of verteporfin [9]. Given these challenges, anti-vascular endothelial growth factor (anti-VEGF) therapy has been proposed as a potential alternative, with studies demonstrating that agents such as bevacizumab, ranibizumab, and aflibercept can reduce choroidal hyperpermeability, promote SRF resolution, and decrease choroidal thickness in chronic CSC (cCSC) [10,11,12]. Among these, aflibercept has shown greater efficacy due to its ability to neutralize VEGF-A, VEGF-B, and placental growth factor (PlGF) [12].

Brolucizumab (Beovu, Novartis Pharma AG, Basel, Switzerland), a next-generation anti-VEGF agent, has garnered attention for its smaller molecular size, higher tissue penetration, and longer duration of action compared to other anti-VEGF drugs [13]. Unlike ranibizumab and aflibercept, brolucizumab consists of a single-chain antibody fragment with a molecular weight of only 26 kDa, allowing for potentially deeper penetration into the retina and choroid. This enhanced bioavailability, combined with its high affinity for VEGF-A isoforms, suggests that brolucizumab may exert a more potent effect on choroidal vasculature, making it a promising therapeutic option for cCSC. However, despite its theoretical advantages, no consensus currently exists on the role of intravitreal brolucizumab in managing cCSC, and clinical data remain limited.

In this study, we aimed to evaluate the efficacy and safety of intravitreal brolucizumab in patients with symptomatic cCSC.

## 2. Materials and Methods

### 2.1. Study Design

This was a single-center, retrospective study from Hanyang University Guri hospital and approved by the institutional review board from both institutes (IRB number: 2025-03-005-001). Retinal specialists offered an off-label treatment with brolucizumab for cCSC in either naïve cases or those with prior poor responses from other treatment modalities, such as PDT, subthreshold micropulse laser (SML), or other anti-VEGF treatments other than brolucizumab. This study was conducted in compliance with the ethical standards outlined in the Declaration of Helsinki. Informed consent about off-label treatment was obtained from all participants.

### 2.2. Participants Characteristics

Patients who visited the ophthalmology clinic between 1 January 2024, and 31 December 2024, were eligible for the study if they had a symptom duration of more than 3 months due to cCSC. cCSC was characterized by RPE changes in the macular region, the presence of SRF in the foveal region persisting for at least three months, and the possible presence of serous pigment epithelial detachment (PED) as observed on OCT. Additionally, fluorescein angiography revealed areas of leakage and/or PED, while ICGA demonstrated abnormal, dilated choroidal vasculature and choroidal vascular hyperpermeability with evident leakage sites. Exclusion criteria included the presence of PNV or polypoidal choroidal vasculopathy (PCV), other retinal diseases other than CSC, a history of any intraocular surgery within 6 months, and patients who refused the off-label treatment.

Best-corrected visual acuity (BCVA) was assessed using Snellen charts and converted to the logarithm of the minimum angle of resolution (logMAR). BCVA values before brolucizumab injection and 6 months after injection were collected. Age, sex, laterality, and previous treatments administered before brolucizumab injection, as well as relevant medical history including hypertension, diabetes mellitus, dyslipidemia, and cardiovascular disease, were thoroughly investigated. Among the enrolled patients, one patient had a history of PDT treatment prior to their presentation at our hospital and subsequent inclusion in this study due to previous treatment failure. This case was included to assess brolucizumab’s efficacy in eyes that had demonstrated partial response or refractoriness to conventional therapies like PDT.

Only one eye per patient was included in the analysis. In cases where both eyes were eligible, the eye with more active or persistent SRF was selected for treatment and evaluation, regardless of laterality. In our cohort, 6 left eyes and 9 right eyes were ultimately included.

All patients showed pachychoroid features with enlarged choroidal vessels. The subfoveal choroidal thickness (SCT) measured at the initial diagnosis (prior to any treatment) was 475.87 ± 107.66 µm, consistent with the pachychoroid spectrum. Detailed inclusion and exclusion criteria, as well as the flowchart of patient recruitment and enrollment, are provided in the Supplementary Materials (Supplementary Table S1 and Supplementary Figure S1).

### 2.3. Intravitreal Brolucizumab Injection

All intravitreal injections were performed using brolucizumab (Beovu^®^, Novartis Pharma AG, Basel, Switzerland), provided in pre-filled syringes containing 6 mg in 0.05 mL. The injections were administered under aseptic conditions following standard protocols. Intravitreal brolucizumab injections were performed to achieve complete resolution of SRF, assessed by OCT. Regarding clinical decision-making, the choice to switch to brolucizumab was primarily based on suboptimal responses to prior therapies, including bevacizumab, aflibercept, PDT, or SML. The decision to initiate brolucizumab was made at the discretion of the treating retina specialists, factoring in persistent SRF, anatomical features consistent with cCSC without PNV, and patient preference after counseling regarding off-label use. For patients who had received prior treatments, brolucizumab injection was specifically initiated if SRF persisted after confirming no satisfactory response for at least 8 weeks from the previous injection. Brolucizumab injections were discontinued if SRF did not recur after treatment, whereas reinjection was performed in cases of recurrence. Patients underwent comprehensive ophthalmic examinations including OCT at scheduled follow-up visits at 1 month and 6 months after the brolucizumab injection. All OCT data after 1 month and 6 months were analyzed. A complete response was defined as the absence of SRF recurrence for at least six months, while a partial response was defined as SRF recurrence within six months. If SRF did not fully resolve during the follow-up period, the corresponding OCT data were excluded from the analysis. The occurrence of intraocular inflammation (IOI), retinal vasculitis, retinal vascular occlusion, endophthalmitis, RPE tears, and retinal detachment after brolucizumab injection was also assessed through comprehensive ophthalmic examinations, including evaluation of clinical signs, wide fundus photography, and OCT.

### 2.4. Retinal Imaging

SS (Swept source)-OCT (DRI OCT-1 Triton, Topcon Corporation, Tokyo, Japan) was used in the study. The SS-OCT images were reviewed and analyzed using an Image Viewer (IMAGEnet 6, version 1.34.1.19417, Topcon Corp., Tokyo, Japan). The central subfield thickness (CST) was measured automatically, with the center of the fovea being automatically positioned (within the central 1-mm diameter circle of the ETDRS grid generated by Topcon software (IMAGEnet 6 Version 1.28.17646; Topcon Corporation, Tokyo, Japan)), and SCT was measured manually from the RPE to the choroidal-scleral interface under the foveal center by using the built-in measurement function of the software. Two examiners (S.H. and Y.U.S.) reviewed all B-scans in the volumetric cube to determine and measure the largest PED across the entire macula, and the height and width of the largest PED were measured manually. In cases of segmentation errors, two examiners independently performed manual corrections, and the average of their measurements was used for analysis. All values were measured on the day of the injection of brolucizumab and 1 month and 6 months after the injection. The presence of SRF was qualitatively evaluated at each time points.

The choroidal vascularity index (CVI, %) was also calculated from subfoveal OCT B-scans using ImageJ software (version 1.54 g). A 1500 μm region centered on the fovea (±750 μm) was binarized using the Niblack thresholding method to differentiate luminal and stromal areas, and CVI was defined as the ratio of the luminal area to the total choroidal area (LA/TCA) [14,15]. All CVI measurements were independently reviewed by two graders (S.H. and Y.U.S.), and the average value was used. To minimize bias, image-based evaluations were performed in a masked manner with respect to patient identity and clinical response.

### 2.5. Statistical Analysis

All values are presented as the mean ± standard deviation. Statistical analysis was performed using SPSS version 22.0 (SPSS, Inc., Chicago, IL, USA), with a *p*-value of <0.05 considered statistically significant.

The Mann–Whitney U test was used for comparing BCVA results before and after brolucizumab injection. Structural changes were evaluated with OCT prior to the brolucizumab injection, at 1 month, and at 6 months after the injection. Changes in CST and SCT after treatment were compared using the Friedman test for paired continuous variables, and two time points were compared using Wilcoxon’s ranked test. CVI before and 6 months after injection was compared using a paired *t*-test.

Effect sizes were calculated using Cohen’s d for paired samples to assess the magnitude of treatment effects, and 95% confidence intervals (CI) were computed through bootstrap resampling to provide estimates of precision. These statistical descriptors were used to complement *p*-values and provide a more comprehensive interpretation of clinical relevance.

## 3. Results

In total, 15 eyes of 15 patients were involved in this study, and there were 7 males (46.7%) and 6 left eyes (40%). The mean age of the patients was 56.53 ± 12.01 years. All patients presented SRF and PED and pachychoroid features with enlarged choroidal vessels, but no drusens were detected. ICGA was available and the results were consistent with cCSC. Ethnicity information was not uniformly recorded, but all patients were of East Asian descent, being treated at a single tertiary hospital in South Korea. One patient had a documented medical history of systemic autoimmune disease: Systemic Lupus Erythematosus (SLE) with hydroxychloroquine use and chronic kidney disease (CKD), while the remaining patients had no notable systemic comorbidities reported in the medical records.

**Figure 1:**
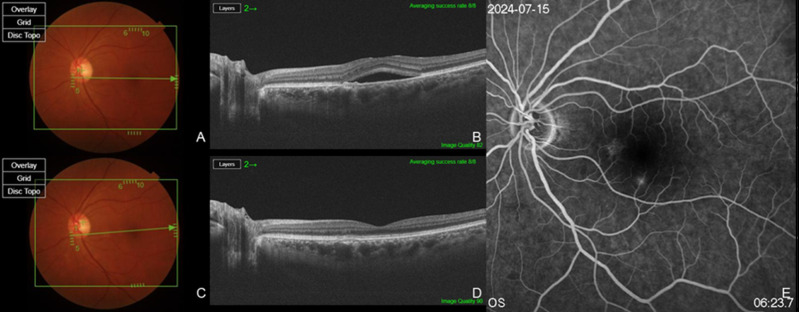
Multimodal retinal imaging in a treatment-naïve patient with chronic central serous chorioretinopathy (cCSC). Baseline fundus photography (**A**) and swept source (SS)-optical coherence tomography (OCT) (**B**) show mild pigment epithelial detachment (PED) and subretinal fluid (SRF) at the macula. At one month after intravitreal injection of brolucizumab, fundus photography (**C**) and SS-OCT (**D**) demonstrate complete resolution of SRF and significant reduction in PED height. Fluorescein angiography (**E**) reveals minimal leakage without evidence of pachychoroid neovasculopathy (PNV) or polypoidal lesions, supporting the diagnosis of uncomplicated cCSC. Subfoveal choroidal thickness (SCT) decreased from 401 μm at baseline (**B**) to 208 μm (**D**) after injection.

**Figure 2:**
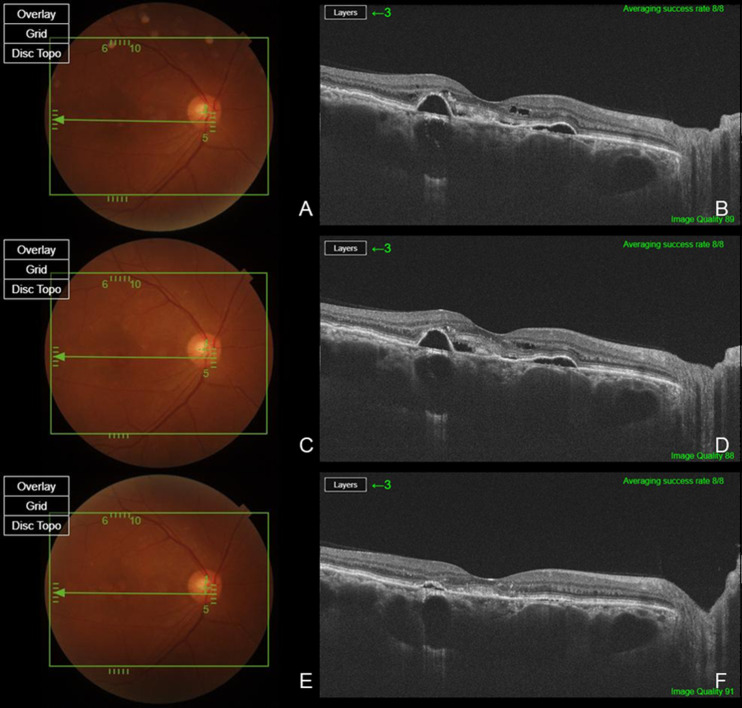
Therapeutic response after switching to brolucizumab in a previously treated patient with bevacizumab. Baseline fundus photography (**A**) and SS-OCT (**B**) demonstrate persistent SRF and PED, despite six prior intravitreal bevacizumab injections. One month after the last bevacizumab injection, SRF and PED remain unchanged, as shown by fundus photography (**C**) and SS-OCT (**D**). Following a switch to intravitreal bolucizumab, fundus photography (**E**) and SS-OCT (**F**) at one month post-injection reveal complete resolution of SRF and significant reduction in PED height, indicating superior anatomical efficacy of brolucizumab in this refractory case. While the SCT value remained nearly unchanged from a baseline of 705 μm (**B**) to 701 μm (**D**) after bevacizumab injection, it decreased to 643 μm (**F**) one month after brolucizumab administration.

Two patients had a history of prior PDT or SML treatment, while eight patients had previously received bevacizumab, and five patients had a history of Aflibercept injections (Figure 1). Six eyes from six patients were treatment-naïve (Figure 2). The total number of injections for all patients was 5.20 ± 4.00, with 3.60 ± 3.83 anti-VEGF injections excluding brolucizumab and 1.60 ± 1.12 brolucizumab injections. Among the 15 patients, 14 patients (93.3%) exhibited complete resolution within six months following brolucizumab injection, while 1 patient demonstrated a partial response. A summary of the types and frequency of anti-VEGF injections, PDT status, and the number of brolucizumab injections in previously treated eyes, as well as the response to brolucizumab in treatment-naïve and previously treated eyes, is presented in Table 1.

To further enhance the interpretability of anatomical and functional outcomes in relation to prior treatment status, we present a comparative summary of key clinical parameter changes between the treatment-naïve and refractory eyes in Table 2. Although formal statistical comparison between groups was not performed due to the limited number of treatment-naïve eyes (n = 1), the naïve patient demonstrated a greater reduction in CST (−127.67 ± 75.51 µm vs. −104.51 ± 78.52 µm) and SCT (−98.33 ± 23.50 vs. −73.94 ± 34.15 µm) compared to the refractory group. This may suggest a potentially more favorable anatomical response in treatment-naïve eyes, although further validation in larger cohorts is needed.

In 14 patients, SRF completely resolved within one month after brolucizumab injection, and this effect persisted for up to six months. In one patient, although SRF significantly decreased compared to baseline, a minimal amount remained. At six months, SRF increased, necessitating reinjection; however, complete resolution was not achieved, and the patient is currently continuing brolucizumab treatment.

BCVA improved in 12 eyes (80.0%) and remained stable in 3 eyes (20.0%), with no cases of deterioration in BCVA. BCVA before brolucizumab injection was 0.34 ± 0.33 in logMAR, and 0.14 ± 0.13, 6 months after brolucizumab injection (*p* = 0.007). The effect size for BCVA in log MAR improvement was −0.81 (95% CI: −0.33 to −0.06), suggesting a large functional gain.

**Figure 3:**
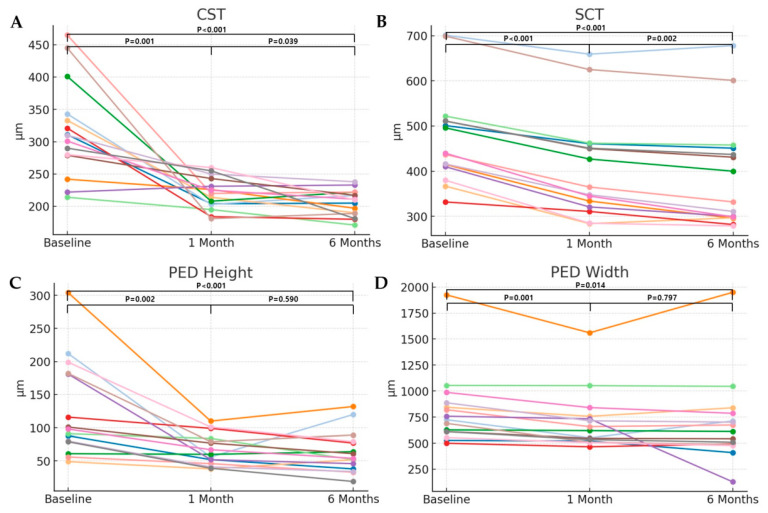
Longitudinal changes in central subfield thickness (CST), SCT, PED height, and PED width following intravitreal brolucizumab injection. CST showed a significant reduction at 1 month after injection compared to baseline, and this reduction was well maintained through 6 months (**A**). SCT also tended to decrease slightly at 1 month post-injection, and this change persisted up to 6 months, although the magnitude of reduction was modest (**B**). PED width did not show remarkable changes in most patients (**D**); however, PED height demonstrated statistically significant decreases at both 1 and 6 months post-injection compared to baseline (**C**). The lines in different colors represent different patients.

CST decreased from 317.13 ± 73.40 μm to 219.93 ± 24.79 μm at 1 month after the brolucizumab injection (*p* = 0.001), and to 205.53 ± 20.17 μm at 6 months after the brolucizumab injection (*p* < 0.001). CST significantly decreased from baseline to 6 months, with a Cohen’s d effect size of –1.52 (95% CI: –2.08 to –0.97), indicating a large anatomical treatment effect. Likewise, SCT decreased from 475.87 ± 107.66 μm to 408.53 ± 114.07 μm at 1 month after the brolucizumab injection (*p* < 0.001), and to 390.13 ± 121.67 μm at 6 months after the brolucizumab injection (*p* < 0.001). This corresponded to a large anatomical effect, with a Cohen’s d effect size of −2.78 (95% CI: −3.33 to −2.22). The height of the PED was 126.47 ± 72.76 μm before brolucizumab injection, and decreased to 66.73 ± 23.79 μm (*p* = 0.002), and 63.33 ± 31.69 μm (*p* < 0.001), at 1 month and 6 months after brolucizumab injection, respectively. The mean PED height significantly decreased from baseline to 6 months, with a Cohen’s d effect size of –1.27 (95% CI: –1.82 to –0.71), indicating a moderate-to-large anatomical response. The width of the PED also decreased from 807.73 ± 350.36 μm to 704.80 ± 283.95 μm (*p* = 0.001), and 691.20 ± 407.22 μm (*p* = 0.014), at 1 month and 6 months, respectively. The mean PED width showed a moderate reduction over the same period, with a Cohen’s d effect size of –0.72 (95% CI: –1.28 to –0.17) (Figure 3).

During the follow-up period, none of the treated eyes exhibited IOI, retinal vasculitis, retinal vascular occlusion, endophthalmitis, RPE tears, or retinal breaks and/or detachment.

**Figure 4:**
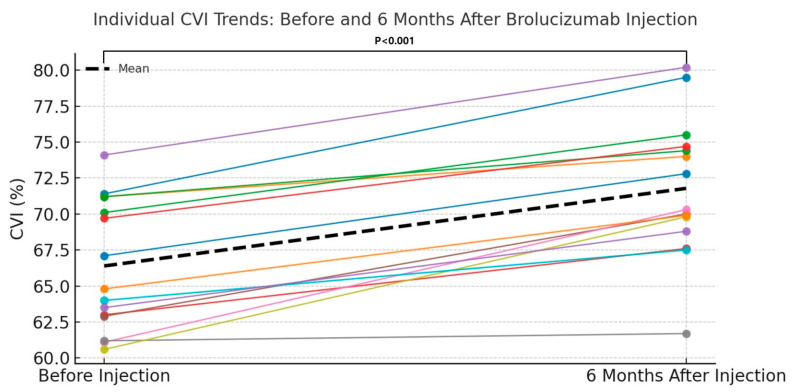
Each colored line represents the CVI trend of an individual patient, while the black dashed line indicates the mean CVI. A general increasing trend is observed in most patients following brolucizumab treatment. The mean CVI showed a statistically significant increase from baseline to 6 months post-injection (*p* < 0.001), suggesting a potential choroidal vascular remodeling effect of brolucizumab in patients with cCSC.

The mean CVI at baseline (pre-injection) was 66.39 ± 4.51, which significantly increased to 71.78 ± 4.82 at 6 months after intravitreal brolucizumab injection. The mean difference in CVI was 5.39 ± 2.39, and this change was found to be statistically significant using a paired t-test (*p* < 0.001) (Table 3). CVI increased significantly, with a Cohen’s d of +2.26 (95% CI: +1.70 to +2.81), indicating a very large vascular remodeling effect. Furthermore, Figure 4 provides a comparative bar graph illustrating individual and mean CVI values before and 6 months after brolucizumab injection.

To enable comparison of anatomical outcomes between brolucizumab and prior anti-VEGF agents, we analyzed response profiles at 1 month following the first injection of other agents (bevacizumab or aflibercept) in patients who later switched to brolucizumab. As summarized in Table 4, anatomical improvements after the initial injection of other anti-VEGF agents were modest. In contrast, brolucizumab resulted in greater mean reductions across all measured parameters, including CST, SCT and PED height and width. These findings suggest enhanced anatomical efficacy of brolucizumab in cases previously unresponsive to standard anti-VEGF therapy.

Furthermore, the CVI, CST, SCT, and BCVA changes were also measured in the untreated fellow eyes to more clearly isolate the effects of brolucizumab from systemic or physiological variations (Table 2, Table 3 and Table 4).

## 4. Discussion

This study evaluated the efficacy and safety of intravitreal brolucizumab in patients with cCSC. The results demonstrated that brolucizumab effectively reduced SRF, CST, SCT, and PED size with a high rate of complete resolution (93.3%) within six months of treatment. Notably, improvements in BCVA were observed in 80% of cases, with no cases of visual deterioration. Also, there were no significant ocular complications such as IOI, retinal vasculitis, or vascular occlusion, supporting the safety profile of brolucizumab in cCSC management.

Interestingly, our study observed not only reductions in SRF, CST and SCT, but also a statistically significant increase in CVI after treatment. This may suggest a degree of choroidal vascular remodeling, potentially reflecting either dilation of large choroidal vessels or reduction in stromal edema. Such changes could be indicative of improved choroidal circulation and fluid dynamics following potent VEGF suppression. These findings support the hypothesis that brolucizumab may exert additional effects beyond VEGF-A inhibition, possibly modulating choroidal structure and RPE function. While the precise mechanism remains to be fully elucidated, our data provide preliminary evidence of brolucizumab’s ability to induce beneficial vascular remodeling in non-neovascular pachychoroid spectrum disease.

To our knowledge, this is one of the first studies to examine these mechanistic implications in cCSC without PNV over a six-month period, thereby addressing a key gap in the literature. Future studies with larger cohorts and multimodal imaging analysis are warranted to further clarify the biological effects of brolucizumab on the choroid and outer retina in cCSC.

PDT with verteporfin was considered a first-line treatment for cCSC, given its high success rates in inducing SRF resolution. Numerous studies have documented the efficacy of half-dose or half-fluence PDT in CSC, with complete fluid resolution in 80–90% of eyes and significant visual improvement in most cases [16]. PDT works via choroidal vascular remodeling: it reduces choroidal congestion and leakage, thereby addressing the root cause of CSC. In the previously mentioned randomized trial, low-fluence PDT vastly outperformed ranibizumab injections [16]. Similarly, other trials comparing PDT to thermal laser or observation consistently show PDT achieving faster and more durable fluid remission. For example, the PLACE trial demonstrated that half-dose PDT was superior to a high-density subthreshold micropulse laser in achieving SRF resolution and vision gains in chronic CSC [17]. Long-term follow-ups indicate that a single PDT treatment can result in long-lasting remission of CSC in a majority of patients [17].

SML is a tissue-sparing laser modality that has been explored as a safer alternative to PDT (which requires intravenous verteporfin and can cause transient RPE atrophy or choroidal ischemia). A micropulse laser can be applied to the macular area without visible burn scars, aiming to stimulate RPE pump function. The efficacy of SML in cCSC has been inconsistent. The PLACE trial found SML to be markedly less effective than PDT: only ~15% of eyes had SRF resolution with micropulse vs. ~67% with PDT at 7–8 months [17]. However, some more recent studies have suggested SML can have a beneficial effect, especially in milder cases or when multiple treatments are applied. A 2023 randomized trial reported that half-dose PDT and yellow 577 nm micropulse were both viable options, with PDT leading to faster anatomic recovery but final visual outcomes that were comparable between the two treatments [18]. Thus, while micropulse laser may eventually improve chronic CSC, it often requires more time or repeat sessions, and its success rate for complete fluid resolution is generally lower than that of PDT.

The clinical utility of anti-VEGF therapy for cCSC has been explored with agents like bevacizumab, ranibizumab, and aflibercept, though the results have been variable [19,20,21,22]. Earlier studies of bevacizumab suggested it may expedite fluid resolution. In a prospective controlled study, Artunay et al. treated cCSC patients with intravitreal bevacizumab and found faster recovery of visual acuity and central thickness compared to observation [19]. All eyes eventually improved, but the bevacizumab group achieved significant VA gain and central foveal thickness reduction as early as 1–3 months [19]. A small randomized trial likewise showed that a single bevacizumab injection led to greater short-term improvement in CSC than no treatment [20]. However, other series have reported that long-term outcomes with bevacizumab may not significantly differ from natural history once the fluid eventually resolves [21]. A large retrospective study of 78 eyes found that ~77% responded anatomically to bevacizumab (complete SRF resolution), while ~23% had refractory fluid; responders experienced significant visual acuity and choroidal thickness improvements [22]. These data indicate that anti-VEGF therapy can benefit a subset of CSC patients, but the effect with first-generation agents is often partial or delayed.

Ranibizumab, a smaller Fab fragment anti-VEGF, has also been tried in cCSC with limited success. A landmark randomized trial by Kim et al. compared ranibizumab (0.5 mg monthly for 3 months) to low-fluence PDT in chronic CSC. After one year, only 12.5% of ranibizumab-treated eyes had complete SRF resolution (most required rescue PDT), versus 88.9% in the PDT group (*p* < 0.001) [16]. Ranibizumab monotherapy yielded inferior anatomic and visual outcomes, underscoring the relatively modest efficacy of conventional anti-VEGF in cCSC when used alone. Aflibercept, a larger fusion protein with high VEGF affinity, has shown somewhat better anatomical outcomes in some studies. In the prospective CONTAIN study (12 eyes), monthly aflibercept for 6 months led to complete fluid resolution in 50% of eyes and significant thinning of the retina and choroid [23]. Although mean VA did not significantly improve over 6 months, half of the patients gained ≥ 1 line and none had major vision loss. Yoon et al. demonstrated in a randomized placebo-controlled study that patients with subacute CSC treated with aflibercept showed significantly greater reductions in CST and superior visual acuity improvement compared to the placebo group [12].

Against this backdrop, our findings with brolucizumab suggest a more pronounced therapeutic effect in cCSC. A prior study has suggested that brolucizumab may also be effective in managing PNV with cCSC [24]. However, unlike previous studies that focused on CSC cases with neovascular complications, our study demonstrates that brolucizumab is also effective in eyes with cCSC without PNV. These findings reinforce the notion that brolucizumab’s potent VEGF-A suppression and deep tissue penetration may provide therapeutic benefits in a broader spectrum of pachychoroid diseases. One investigation evaluated the efficacy of brolucizumab in cCSC without PNV, but it was conducted on five eyes from four patients with a follow-up period of one month [25]. The findings suggest that brolucizumab may be effective in rapidly reducing persistent macular fluid in cCSC without CNVM, which aligns with our study. However, longer follow-up is necessary to assess recurrence rates and long-term safety. Therefore, our study included a larger cohort of 15 eyes from 15 patients with a follow-up period of six months. In our series, the majority of eyes achieved complete or near-complete SRF resolution after a single injection, an outcome that typically required PDT in prior studies. Brolucizumab is a uniquely engineered anti-VEGF agent that may be particularly well-suited to address the choroidal vascular hyperpermeability in CSC. It is a humanized single-chain antibody fragment targeting VEGF-A, distinguished by its small molecular size of 26 kDa [26]. By comparison, ranibizumab is ~48 kDa and aflibercept ~115 kDa. This smaller size and lack of an Fc region confer several pharmacologic advantages. First, a 6 mg dose of brolucizumab contains a very high molar quantity of drug—approximately 10–12 times the molar dose of aflibercept and ~20 times that of ranibizumab [27]. This high molar concentration enables more complete and sustained VEGF-A blockade in ocular tissues. Second, brolucizumab’s tiny molecular footprint allows deeper tissue penetration. Preclinical studies indicate it can more effectively permeate the retina and RPE-choroid complex than larger molecules [28]. In the context of CSC, where the pathology lies at the level of the choroid and RPE, this superior penetration might translate to greater suppression of choroidal vascular leakage. It may explain why brolucizumab achieved rapid SRF resolution in our patients; the drug likely reached the choriocapillaris and RPE more efficiently to reduce choroidal hyperpermeability. Another key property is duration of action. Brolucizumab was designed for durability, as evidenced by its performance in neovascular age-related macular degeneration (AMD) trials. Over half of brolucizumab-treated nAMD eyes were maintained on a 12-week dosing interval with non-inferior vision outcomes compared to 8-week aflibercept [13]. Furthermore, brolucizumab showed superior drying on OCT (less intraretinal and subretinal fluid) than aflibercept at similar time points. This suggests that brolucizumab provides more robust and lasting resolution of exudation. In cCSC, improved durability could be advantageous: cCSC often requires prolonged management, and a longer-acting agent might keep the macula dry for extended periods with fewer injections. In our pilot, 14 out of 15 patients achieved complete SRF resolution after a single brolucizumab injection, with only one requiring reinjection during the six-month follow-up. Alongside the meaningful and sustained reduction in SCT observed throughout this period, these findings suggest that brolucizumab may exert a more durable therapeutic effect on choroidal vasculature compared to conventional anti-VEGF agents. Interestingly, despite reductions in SRF and SCT, a relative increase in CVI was observed at 6 months post-injection. This suggests underlying choroidal vascular remodeling, potentially through vascular dilation or stromal thinning. As CVI represents the ratio of luminal area to total choroidal area, its increase may reflect enlargement of large-caliber vessels, such as those in the Haller layer, aligning with characteristics of the pachychoroid spectrum [15,29]. Alternatively, resolution of stromal edema or inflammation may reduce stromal volume, making vascular structures appear more prominent. These findings imply that brolucizumab may exert additional effects on the choroid beyond VEGF-A suppression alone. Notably, a previous report documented choroidal vascular remodeling following brolucizumab loading in AMD patients—a change not observed with aflibercept [30]. Similarly, the reduction in SRF in cCSC may correlate with vascular remodeling of choroid triggered by brolucizumab’s high tissue penetrance and potent VEGF inhibition. The extended tissue residence and potent VEGF suppression could reduce the tendency for fluid recurrence, though longer follow-up is needed to verify recurrence rates. Additionally, there is speculation that anti-VEGF treatment could transiently improve RPE barrier function or promote fluid egress. For instance, resolution of subretinal fibrin in a CSC case after brolucizumab was attributed to increased RPE pump activity and fibrin absorption following VEGF blockade [31]. While the exact mechanistic interplay between VEGF and cCSC’s pathophysiology is not fully understood, our results imply that maximal VEGF-A inhibition by brolucizumab can favorably shift the equilibrium toward fluid resorption in cCSC. However, it is important to note that the accuracy of CVI measurements using (version 1.54k)binarization methods like the Niblack threshold in ImageJ can be influenced by intrinsic optical properties of the choroid, including directional reflectance and diffuse scattering. These factors may affect the differentiation between luminal and stromal areas on OCT, especially under varying imaging conditions. As such, our CVI results should be interpreted with caution, acknowledging the influence of these reflectance characteristics on the segmentation process [14,15]. Future studies incorporating directional OCT or adaptive optics may help refine CVI quantification and improve the robustness of these metrics.

This study has several limitations. The retrospective nature and the absence of a control group limit the ability to draw definitive conclusions. In particular, without comparative group, it is difficult to determine whether the observed anatomical improvements are solely attributable to brolucizumab, as prior studies have demonstrated varying degrees of success with standard treatments such as aflibercept and PDT [16,17,18]. To partially address this limitation, we additionally analyzed the anatomical changes observed following previous intravitreal injections prior to brolucizumab treatment (Table 4). This approach allowed us to use each patient’s own pre-treatment response as a surrogate control for comparison. Furthermore, we have included an analysis of the untreated fellow eyes, which provides a valuable internal control to help mitigate the absence of a dedicated control group (Table 2, Table 3 and Table 4). Nonetheless, future studies with randomized controlled designs are essential to validate these findings in a more rigorous setting.

Moreover, the heterogeneity of our cohort, particularly that 60% of patients had received previous anti-VEGF agents or PDT, further complicates the attribution of observed effects exclusively to brolucizumab. These prior treatments may have contributed to residual anatomical responses or influenced treatment dynamics, making it challenging to isolate the drug’s true efficacy. Stratified or treatment-naïve cohorts in future studies may help to address this limitation. To overcome this limitation, we stratified patients into treatment-naïve and refractory groups for further analysis (Table 2).

In addition, optical confounders such as media opacities or variations in OCT signal strength may have affected image-based measurements like CVI. Although signal strength was not objectively quantified, all OCT scans were obtained by trained technicians using standardized protocols, and poor-quality images with motion artifacts or media opacity interference were excluded from analysis.

Visual acuity was initially assessed using Snellen charts due to clinical workflow constraints, but all values were subsequently converted to logMAR for statistical analysis. While this approach may reduce measurement precision, future studies should adopt logMAR-based charts from the outset to ensure greater accuracy and consistency in research settings.

The short follow-up period and small sample size limit assessment of long-term efficacy, durability, and recurrence. However, the primary goal of this pilot study was to provide initial, real-world evidence of brolucizumab’s effect in preventing short-term recurrence in patients who had previously shown poor responses to other therapies. Therefore, the six-month follow-up was deemed sufficient to address this specific objective. Nonetheless, prospective trials with larger cohorts and longer follow-up periods are needed to directly compare brolucizumab with other anti-VEGF agents and explore its potential in combination therapy, particularly with PDT, which may provide synergistic benefits in cCSC.

Although no cases of IOI, retinal vasculitis, or vascular occlusion were observed in our cohort, it is important to acknowledge these known safety concerns with brolucizumab. Post-marketing surveillance and clinical trial data have reported such adverse events in other disease contexts, particularly in neovascular AMD. The absence of these complications in our study may reflect the limited sample size or differences in disease pathology. Nonetheless, we recommend cautious use of brolucizumab in cCSC, with appropriate informed consent and close post-injection monitoring [32].

Furthermore, a critical consideration for the widespread adoption of brolucizumab for cCSC lies in its cost–benefit profile. Currently, brolucizumab is used off-label for cCSC in South Korea, meaning it is not covered by health insurance reimbursement. This lack of reimbursement places a significant economic burden on patients, which may limit access to this potentially effective therapy despite its clinical benefits. Therefore, further dedicated research focusing on the cost-effectiveness of brolucizumab for cCSC is essential to fully understand its value in real-world clinical practice. Should brolucizumab receive insurance coverage for cCSC in the future, it would substantially alleviate patients’ financial strain and facilitate broader access to this innovative treatment.

In this study, one of the fifteen patients had a history of systemic disease such as SLE and CKD. These systemic conditions can influence circulating levels of inflammatory mediators and VEGF, which are implicated in cCSC pathophysiology [33]. This suggests that such comorbidities could affect a patient’s response to anti-VEGF therapy like brolucizumab. However, due to the small sample size and the fact that only one patient had these specific conditions, it is difficult to accurately determine the extent of their influence on treatment outcomes. Therefore, larger, controlled studies are needed to investigate the impact of these comorbidities on the efficacy of anti-VEGF treatments in cCSC patients.

In summary, brolucizumab offers a novel and potentially superior therapeutic option for managing cCSC by reducing choroidal thickness and promoting subretinal fluid resolution. While further investigation is required, these findings suggest promising efficacy beyond that of conventional anti-VEGF therapies.

## 5. Conclusions

In conclusion, this study suggests that brolucizumab may be a promising treatment option for cCSC without PNV, particularly in patients who have not responded to other anti-VEGF agents. Given the lack of standardized treatment protocols for cCSC, these findings provide valuable real-world evidence supporting the use of brolucizumab as a viable therapeutic option.

## Figures and Tables

**Table 1:** Summary of the types and frequency of anti-VEGF injections, PDT status, and the number of brolucizumab injections in previously treated eyes, as well as the response to brolucizumab in treatment-naïve and previously treated eyes.

Patient Number	Previous PDT or SML	Previous Bevacizumab	Previous Aflibercept	No. of Previous Injection	No. of Brolucizumab Injection	Response to Brolucizumab
1	No	Yes	Yes	6	1	Complete
2	No	Yes	No	6	1	Complete
3	No	Yes	No	9	1	Complete
4	No	Yes	Yes	8	2	Complete
5	No	No	No	0	1	Complete
6	No	No	Yes	1	1	Complete
7	Yes	Yes	Yes	6	3	Complete
8	No	Yes	Yes	8	1	Complete
9	No	Yes	No	1	5	Complete
10	Yes	Yes	No	9	2	Partial
11	No	No	No	0	1	Complete
12	No	No	No	0	1	Complete
13	No	No	No	0	1	Complete
14	No	No	No	0	1	Complete
15	No	No	No	0	2	Complete

Abbreviations: Anti-VEGF—anti-vascular endothelial growth factor; PDT—photodynamic therapy; SML—subthreshold micropulse laser.

**Table 2:** Changes in key anatomical and functional parameters between treatment-naïve and refractory eyes from baseline to 6-month follow-up.

Patient Number	Response to Brolucizumab	CST Change (µm)		SCT Change (µm)	
		Treated Eye	Fellow Eye	Treated Eye	Fellow Eye
Naïve (6)	Complete	−127.67 ± 75.51	−0.37 ± 46.87	−98.33 ± 23.50	+0.21 ± 41.01
Refractory (9)	Varies (1 case: partial response)	−104.51 ± 78.52	−0.14 ± 31.12	−73.94 ± 34.15	−0.09 ± 21.42
Patient number	Response to brolucizumab	BCVA Change (logMAR)		PED height Change (µm)	PED width Change (µm)
		Treated eye	Fellow eye		
Naïve (6)	Complete	−0.12 ± 0.10	−0.01 ± 0.87	−59.0 ± 43.15	−110.33 ± 75.88
Refractory (9)	Varies (1 case: partial response)	0.23 ± 0.12	0.02 ± 1.75	−68.45 ± 59.76	−112.57 ± 217.28

All continuous data are expressed as mean values ± standard deviation (range). All values represent change from baseline to the 6-month follow-up. Abbreviations: CST—central subfield thickness; SCT—subfoveal choroidal thickness; PED—pigment epithelial detachment; BCVA—best-corrected visual acuity.

**Table 3:** Comparison of choroidal vascularity index (CVI) before and 6 months after intravitreal brolucizumab injection in patients with chronic central serous chorioretinopathy.

Patient Number	CVI Before Brolucizumab Injection (%)		CVI, 6 Month Brolucizumab Injection (%)		*p*-Value (Treated Eye: 1 mo vs. 6 mo)
	Treated Eye	Fellow Eye	Treated Eye	Fellow Eye	
1	71.4	68.4	79.5	68.1	
2	71.2	69.1	74.0	68.4	
3	71.2	69.2	74.4	69.8	
4	63.0	65.0	67.6	65.1	
5	74.1	70.4	80.2	70.1	
6	62.9	62.8	70.0	63.0	
7	61.1	61.4	70.3	61.1	
8	61.2	61.0	61.7	60.8	
9	60.6	60.9	69.8	60.1	
10	64	61.8	67.5	62.1	
11	67.1	65.4	72.8	65.8	
12	64.8	60.1	69.9	60.0	
13	70.1	64.8	75.5	65.9	
14	69.7	68.9	74.7	68.8	
15	63.5	62.1	68.8	62.5	
Mean	66.39 ± 4.51	64.75 ± 3.61	71.78 ± 4.82	64.77 ± 3.64	<0.001

All continuous data are expressed as mean values ± standard deviation (range).

**Table 4:** Comparative anatomical changes at 1 month after first other anti-VEGF vs. brolucizumab injection.

	Changes 1 Month After Other Anti-VEGF		Changes 1 Month After Brolucizumab Injection		*p*-Value (Treated Eye)
	Treated Eye	Fellow Eye	Treated Eye	Fellow Eye	
CST change (µm)	14.79 ± 5.90	1.01 ± 3.14	107.85 ± 84.56	0.91 ± 8.41	<0.001
SCT change (µm)	27.31 ± 22.31	0.98 ± 9.54	61.14 ± 26.42	1.24 ± 10.18	<0.001
PED height change (µm)	24.72 ± 23.77		79.01 ± 78.24		<0.001
PED width change (µm)	42.03 ± 36.22		121.42 ±125.49		<0.001

All continuous data are expressed as mean values ± standard deviation (range). All values represent change from baseline to the 6-month follow-up. Abbreviations: CST—central subfield thickness; SCT—subfoveal choroidal thickness; PED—pigment epithelial detachment; Anti-VEGF—anti-vascular endothelial growth factor.

## Data Availability

The datasets generated and/or analyzed during the current study are not publicly available due to ethical and patient confidentiality restrictions imposed by the institutional review board. However, the data may be available from the corresponding author upon reasonable request and with appropriate institutional approvals.

## Supplementary Materials

The following supporting information can be downloaded at: https://www.mdpi.com/article/10.3390/jpm15090409/s1, Figure S1: Flowchart of Patient Recruitment and Enrollment; Table S1: Inclusion and Exclusion Criteria for the Study Population.
