# Supplementary material for: Efficacy of Intravitreal Brolucizumab for Chronic Central Serous Chorioretinopathy: A Pilot Study
Source: J Pers Med. 2025 Sep 2;15(9):409. doi: 10.3390/jpm15090409 (PMC12470493; doi:10.3390/jpm15090409)

Efficacy of Intravitreal Brolucizumab for Chronic Central Serous Chorioretinopathy: A Pilot Study

Supplementary Table S1. Inclusion and Exclusion Criteria for the Study Population

	Inclusion Criteria	Exclusion Criteria
Diagnosis	Patients with symptomatic cCSC<br><br>Persistent foveal SRF for at least 3 months<br><br>RPE changes in the macular regi on on OCT<br><br>Leakage/PED on fluorescein angi ography<br><br>Abnormal, dilated choroidal vasc ulature and hyperpermeability on ICGA	Presence of PNV or PCV<br><br>Other retinal diseases besides cCSC
Patient History	Symptom duration of more than 3 months	History of any intraocular surgery within 6 months<br><br>Patients who refused off-la bel treatment
Previous Treatments	Naïve cases or cases with poor r esponses to prior treatments (e.g., PDT, SML, other anti-VEGF age nts)	
Study Participation	Only one eye per patient was in cluded, with the more active or persistent SRF selected	

Abbreviations: cCSC; chronic central serous chorioretinopathy, SRF; subretinal fluid, OCT ; optical coherence tomography, PED; pigment epithelial detachment, ICGA; indocyanine green angiography, PDT; photodynamic therapy, SML; subthreshold micropulse laser, Anti-VEGF; anti-vascular endothelial growth factor, PCV; polypoidal choroidal vasculopathy, PNV; pachychoroid neovasculopathy

Supplementary Figure S1. Flowchart of Patient Recruitment and Enrollment

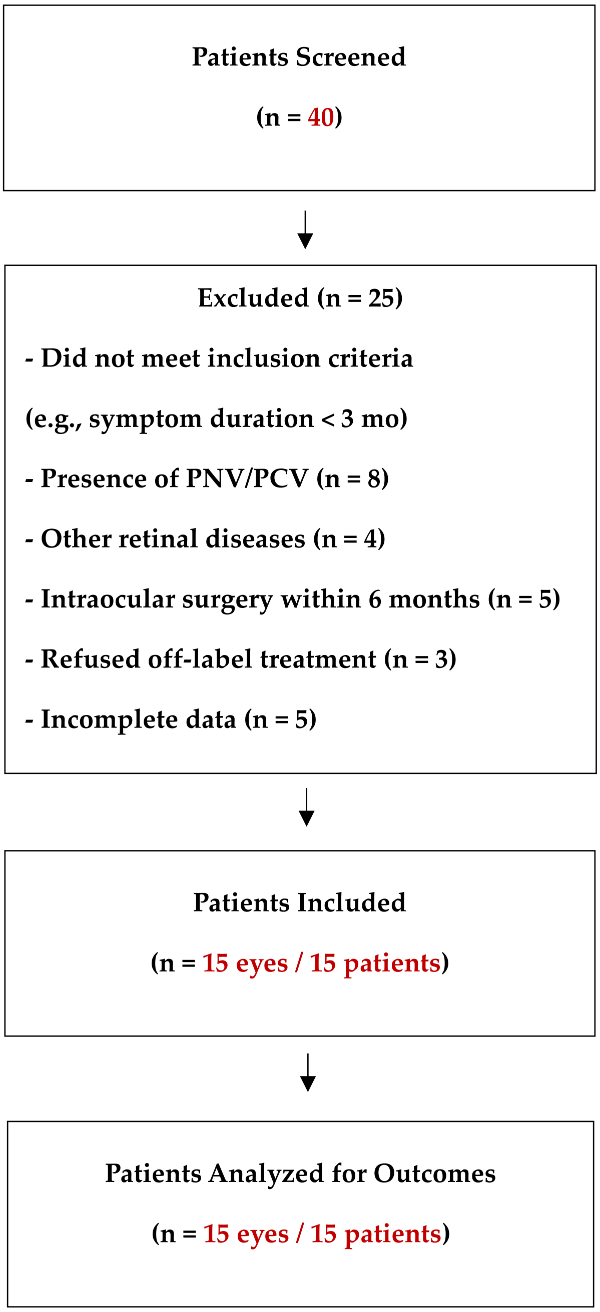

Supplement: Supplementary file 1 [file jpm-15-00409-s001.zip › jpm-3734151-supplementary.pdf]
